# Evaluation of Glucose 6-Phosphate Dehydrogenase, Pyruvate Kinase, and New Generation Inflammation Biomarkers in Prolonged Neonatal Jaundice

**DOI:** 10.3390/medicina60091491

**Published:** 2024-09-12

**Authors:** Omer Okuyan, Seyma Dumur, Neval Elgormus, Hafize Uzun

**Affiliations:** 1 Department of Pediatrics, Medicine Hospital, Faculty of Medicine, Istanbul Atlas University, 34408 Istanbul, Turkey; dmemhs@gmail.com; 2 Department of Medical Biochemistry, Faculty of Medicine, Istanbul Atlas University, 34408 Istanbul, Turkey; seyma_dumur@hotmail.com; 3 Department of Microbiology, Faculty of Medicine, Istanbul Atlas University, 34408 Istanbul, Turkey; neyelgormus@yahoo.com

**Keywords:** prolonged jaundice, breast milk, glucose 6-phosphate dehydrogenase, pyruvate kinase, systemic immune inflammation index, neutrophil-to-lymphocyte ratio

## Abstract

*Background and Objectives*: To evaluate the clinical findings of glucose 6-phosphate dehydrogenase (G6PD) and pyruvate kinase (PK) deficiency in prolonged jaundice and to determine whether the systemic immune inflammation index (SII), neutrophil-to-lymphocyte ratio (NLR), and platelet-to-lymphocyte ratio (PLR) can be used in the diagnosis of neonatal prolonged jaundice. *Materials and Methods*: Among full-term neonates with hyperbilirubinemia who were admitted to Medicine Hospital between January 2019 and January 2024 with the complaint of jaundice, 167 infants with a serum bilirubin level above 10 mg/dL, whose jaundice persisted after the 10th day, were included in this study. *Results*: G6PD activity was negatively correlated with NLR, SII, age, and hematocrit (Hct). There was a weak negative correlation between G6PD and NLR and a moderate negative correlation between G6PD activity and SII when adjusted for age and Hct. PK activity showed no significant correlation with G6PD, NLR, PLR, SII, age, and Hct. A linear relationship was observed between G6PD activity and SII and NLR. *Conclusions*: NLR and SII can be easily calculated in the evaluation of prolonged jaundice in G6PD deficiency has a considerable advantage. NLR and SII levels may contribute by preventing further tests for prolonged jaundice and regulating its treatment. It may be useful to form an opinion in emergencies and in early diagnostic period.

## 1. Introduction

At least two-thirds of newborns become clinically jaundiced in the first week of life [1]. Prolonged jaundice is defined as a bilirubin level above 10 mg/dL on the 14th day after birth in a term baby and on the 21st day in a premature baby [2,3]. The most common cause of prolonged jaundice is breast milk jaundice, but it is important because it can also be a symptom of a serious underlying disease [4]. The most important step in the follow-up of these babies is to determine the etiology.

In babies with prolonged jaundice, a detailed history should be taken, and a detailed physical examination should be performed. Then, as initial tests, direct–indirect bilirubin levels, mother–baby blood groups, a direct Coombs test, a complete blood count, a peripheral smear, the glucose 6-phosphate dehydrogenase (G6PD) enzyme level, thyroid function tests [thyroid stimulating hormone (TSH), thyroxine, (free T4)], urinalysis, a urine culture, and reducing substance in urine should be investigated [2]. Due to mutations in the G6PD gene, more than 150 protein variants and varying rates of enzyme activity related to these variants have been reported. Due to mutations in the G6PD gene, more than 150 protein variants and varying rates of enzyme activity related to these variants have been reported. The frequency varies among societies around the world. Turkey is in a location where G6PD variant genes causing severe hemolysis and jaundice are endemic [5]. The World Health Organization (WHO) recommends that all newborn babies should be screened in this respect in regions where the prevalence of G6PD enzyme deficiency exceeds 3–5% or where deficiency is seen mostly in male babies [6].

Erythrocyte enzyme defects involved in the etiology of neonatal jaundice are G6PD, pyruvate kinase (PK), 5’ nucleotidase, glucose phosphate isomerase, glutathione synthetase, and fructokinase. G6PD deficiency is the most common enzyme deficiency and shows X-dependent transition. G6PD deficiency is more common in male babies. Clinical findings may vary. It presents with jaundice because of hemolysis that develops in babies who are under oxidant stress or who have acidosis, hypoglycemia, or an infection during the neonatal period. Jaundice usually develops between 24 and 72 h [7,8,9,10]. Another important enzyme deficiency is PK deficiency, which is an autosomal recessive inheritance that causes hemolytic jaundice in the neonatal period. A diagnosis can be made using enzyme determination in patients with hemolytic hyperbilirubinemia [10].

The role of inflammation in the etiology of prolonged neonatal jaundice is not fully understood. The cause of the inflammation is unknown, although many mechanisms have been implicated, including immunological, viral, ischemic, and genetic mechanisms. Levels of blood-based systemic inflammation markers (complete blood count (CBC), hemoglobin, neutrophil-to-lymphocyte ratio (NLR), erythrocyte distribution width, etc.), which are considered prognostic indicators for many diseases, have been investigated [8,11,12].

New-generation inflammation markers have been studied in limited numbers in neonatal prolonged jaundice. In the literature search, no article was found in which new-generation inflammatory markers, such as systemic immune inflammation index (SII), NLR, and platelet-to-lymphocyte ratio (PLR) were studied in G6PD and PK deficiency in neonatal prolonged jaundice. The aim of this study was to evaluate the clinical findings of G6PD and PK deficiency in prolonged jaundice. At the same time, the aim is to determine whether SII, NLR, and PLR can be used in addition to conventional CBC parameters in the diagnosis of neonatal prolonged jaundice. We also aimed to evaluate the relationship between G6PD and PK deficiency and new-generation inflammatory markers (SII, NLR, PLR) in neonatal prolonged jaundice.

## 2. Materials and Methods

### 2.1. Study Design and Participants

Ethical approval of this study was obtained by the Non-Interventional Ethics Committee of the Medical Faculty of Istanbul Atlas University (26 October 2022; No: E-22686390-050.99-21197). This study was performed in accordance with the Helsinki Declaration, and informed consent was obtained from the families of all patients prior to their inclusion in this study.

**Figure 1:**
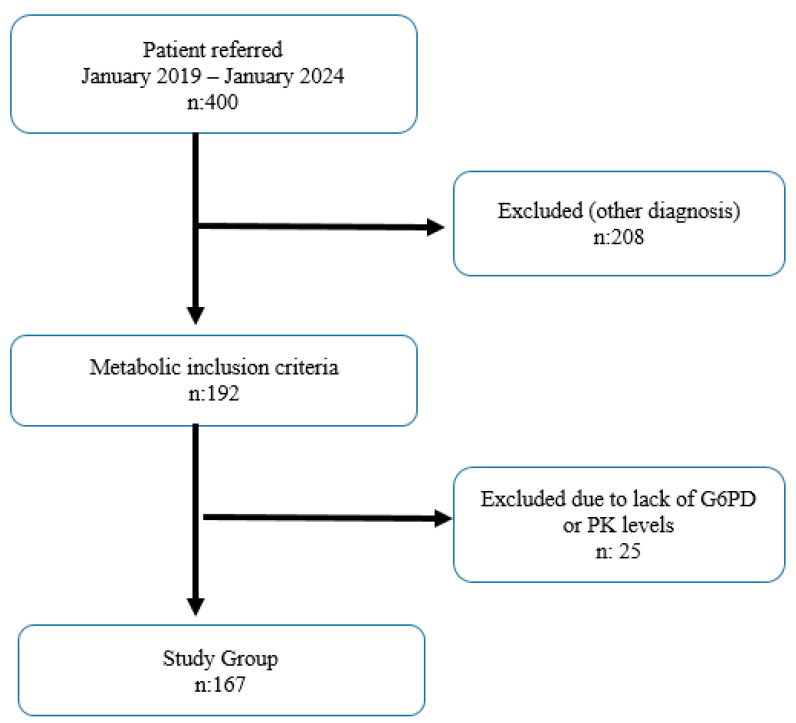
Flowchart of the selection of cases.

In full-term neonates with hyperbilirubinemia who were admitted to Medicine Hospital between January 2019 and January 2024 with the complaint of jaundice, 167 infants with a serum bilirubin level above 10 mg/dL, whose jaundice persisted after the 10th day, were included in this study. A flowchart of the selection of cases is shown in Figure 1. A detailed history was taken from the families of infants with prolonged jaundice. The age of the cases was calculated in days, and the day of birth was accepted as day zero. The day of onset of jaundice was determined as the day the family first noticed jaundice. Newborns with jaundice above physiologic values and prolonged jaundice with no other cause to explain it were included in this study. In the postnatal period, the day of the onset of jaundice, first feeding, time of first urination, first fecal output, and complaints were questioned. Newborns diagnosed with prolonged jaundice with total bilirubin levels above 10 mg/dL were followed in the clinic. Clinically, pathologic findings on physical examination were evaluated. Other systemic examinations were performed in terms of activity, skin color, neonatal reflexes, and focus of infection.

In our clinic, phototherapy and exchange transfusion decisions were made according to the bilirubin threshold values recommended by the American Academy of Pediatrics (AAP) [13]. The decision to discharge the patients was made by the research doctor, according to Nelson Texts icteric neonatal discharge protocol [1].

Breast milk jaundice was diagnosed by exclusion in infants who were exclusively breastfed and older than 14 days with no other identifiable pathogenic factors for jaundice.

### 2.2. Inclusion Criteria

(i) Patients with prolonged jaundice exceeding 15 days and total bilirubin values exceeding 10 mg/dL; (ii) patients who appear icteric on physical examination during routine controls; (iii) patients with hemolytic anemia and negative direct Coombs; (iv) patients who attended regularly for up to 3 months, as patients were invited for the first 6 monthly check-ups; (v) prolonged jaundice was differentiated by looking at direct and indirect bilirubin, and those who were negative by measuring direct Coombs were included; and (vi) those with TSH levels within normal limits were included.

### 2.3. Exclusion Criteria

Premature infants, infants undergoing phototherapy and blood exchange, and infants with congenital malformations, congenital infections, bacterial infection, hypoxic-ischemic encephalopathy, respiratory distress, neonatal hemolytic disease, sepsis, and transfusion exchange were excluded from this study. The patients whose mothers had hypertensive, renal, hepatic, or hematologic diseases, were taking any medications, or were tobacco users were excluded. Patients with thyroid stimulating hormone (TSH) levels higher than 20 mU/L were considered to have congenital hypothyroidism and excluded from this study. Direct Coombs test positivity was accepted as one of the diagnostic criteria in Rh incompatibility, whereas direct Coombs test positivity was not required in ABO incompatibility. Since Rh incompatibility may result in prolonged jaundice in neonates, we excluded patients with direct Coombs positivity. 

#### 2.3.1. Laboratory Assessments

Venous blood samples were taken from all patients at the time of admission. For CBC, 0.5–2 mL blood was drawn into purple capped ethylenediaminetetraacetic acid (EDTA) tubes and measured in an automatic blood count device (Sysmex XN 1000, Roche Diagnostics GmbH, Mannheim, Germany) within 1 h at the latest. The NLR and PLR were calculated as neutrophil count (10^3^ cells/µL)/lymphocyte count (10^3^ cells/µL) and platelet count (10^3^/µL)/lymphocyte count, respectively. The SII was calculated as (platelet count × neutrophil count)/lymphocyte count [11]. Reticulocytes were performed manually using Brilliant Cresyl blue with a correction formula, and routine biochemical parameters were measured in an autoanalyzer (ARCHITECT c8000 Abbott, Holliston, MA, USA).

#### 2.3.2. Measurement of Glucose 6-Phosphate Dehydrogenase (G6PD) Activity

The G6PD activity in whole blood was measured with G6PD kits (Ben Srl Biochemical Enterprise, Milan, Italy) that used a spectrophotometric assay on Roche Cobas c 501, following the manufacturer’s instructions.

#### 2.3.3. Measurement of Pyruvate Kinase (PK) Activity

The PK activity in whole blood was measured with pyruvate kinase kits (Ben Srl Biochemical Enterprise, Milan, Italy) that used a spectrophotometric assay on Roche Cobas Mira, following the manufacturer’s instructions.

### 2.4. Statistical Analysis

Statistical Package for the Social Sciences version 29.0 software package for Windows (IBM Corp., Armonk, NY, USA) was used for data evaluation and analysis. Jamovi 2.3.18 was used to create figures. Categorical variables are presented as frequencies (n) and percentages (%), and numerical variables are presented as mean ± standard deviation and median (25. percentile–75th percentile). The Kolmogorov–Smirnov test was applied for normality analysis. The Mann–Whitney U test was used to compare continuous variables between two independent groups. The Spearman correlation and linear regression analysis were used to determine the association between two continuous variables. Also, a partial correlation was used to show the relationship between two continuous variables while controlling for the effect of other continuous variables. A receiver operating characteristic curve (ROC) analysis was conducted to determine cut-off values, sensitivity, and specificity. A value of *p* < 0.05 was accepted as statistically significant.

## 3. Results

The mean age was 31 ± 6 days, and 63.5% of the patients were male. All patients had a negative direct Coombs test, negative urine culture, and normal abdominal USG. All patients were breastfed, and 12 patients (7.2%) were also receiving formula. ABO incompatibility was present in 6% (n:10), Rh incompatibility was present in 9% (n:15), and reducing substance was present in 16.25% (n:27) of the patients. Seven patients (4.2%) were hospitalized, and the median hospitalization median was 1 (1–1) day. The median day of jaundice onset was 2 (1–2) days. The median time to fall below TB = 8 was 80 (80–80) days. The mean gestational week of the patients was 37 ± 1 weeks (Table 1).

Table 2 shows the laboratory results of the patients. The WBC median was 9110 (7910–11,100), Hct median was 37.1 (32.5–41.4), PLT median was 328 (277–400), NLR median was 0.3 (0.21–0.37), PLR median was 55.38 (43.05–70.76), and SII median was 95.73 (64.61–125.96) (Table 2). 

There was no statistically significant difference in the NLR, PLR, SII, G6PD, and PK values according to gender, hospitalization status, formula use, ABO incompatibility, Rh incompatibility, or urine-reducing substance (Table 3).

**Figure 2:**
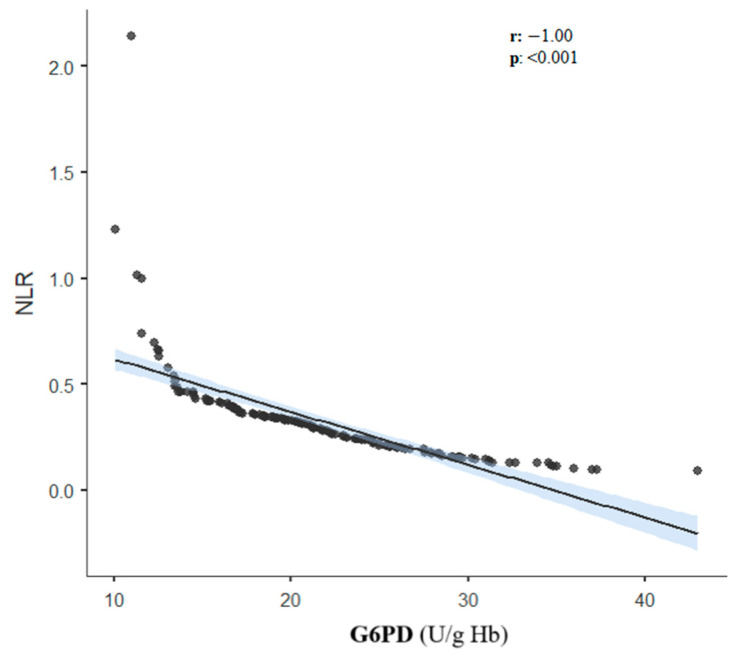
Correlation between G6PD activity and NLR. The black line is the regression line and the blue area is the confidence interval of regression line.

**Figure 3:**
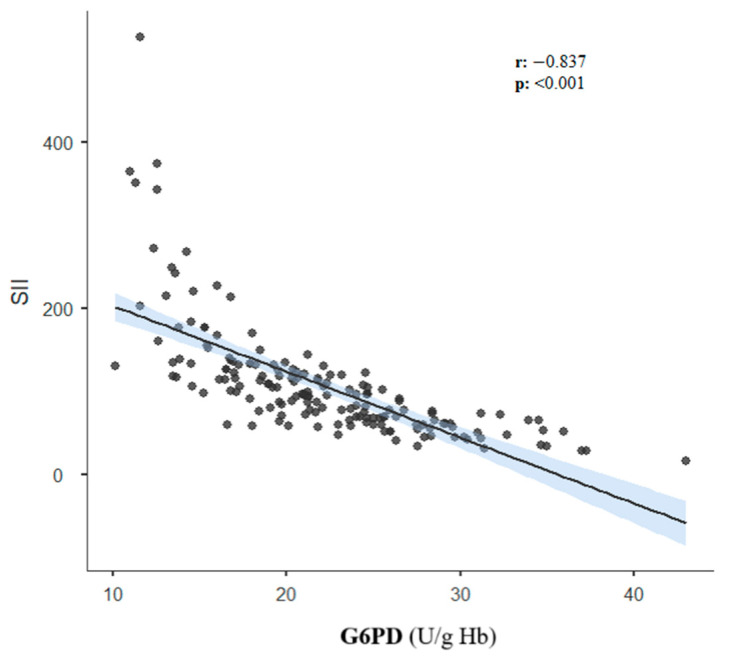
Correlation between G6PD activity and SII. The black line is the regression line and the blue area is the confidence interval of regression line.

There was a perfect negative correlation between NLR and G6PD (r: −1; *p* < 0.001; Figure 2) and a strong negative correlation between SII and G6PD (r: −0.837; *p* < 0.001, Figure 3). There was a weak correlation between age and Hct and age and G6PD (r: 0.296; *p* < 0.001 and r: −0.285; *p* < 0.001, respectively). There was no significant correlation between G6PD and PK (r: 0.00; *p*: 0.960). There was no significant correlation between PK and NLR, PLR, SII, age, and Hct (Table 4). Furthermore, when adjusted for age and Hct, there was a weak negative correlation between G6PD and NLR (r: −0.212; *p*: 0.006) and a moderate negative correlation between G6PD and SII (r: −0.470; *p* < 0.001) (Table 5).

According to the results of linear regression analysis, a 1-unit increase in NLR decreased G6PD by 1.407 units when adjusted for age and Hct (unstandardized (B): −1.407 (−2.411/−0.402); *p*: 0.006). When adjusted for age and Hct, a 100-unit increase in SII decreased G6PD by 2.581 units (unstandardized (B): −2.581 (−3.331/−1.831); *p* < 0.001). The R square of model 1 with NLR, age, and Hct was 0.198, while the R square of model 2 with SII, age, and Hct was 0.346 (Table 6).

**Figure 4:**
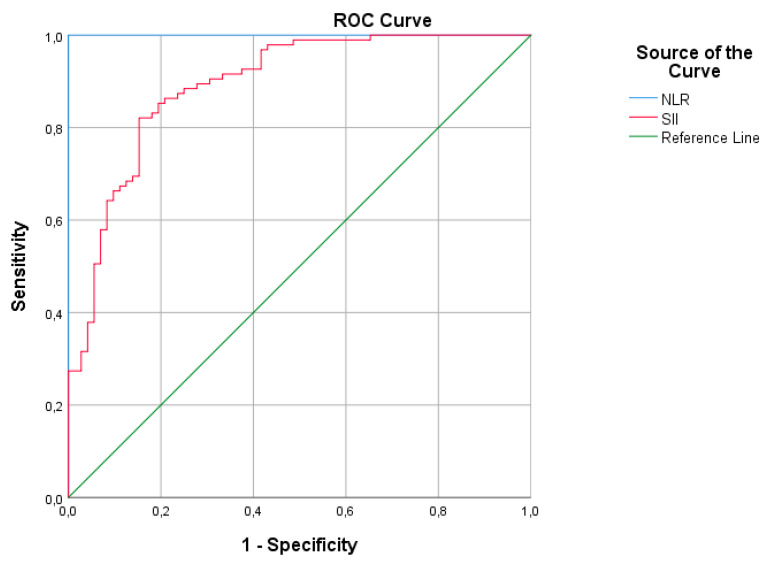
ROC analysis graphic of G6PD activity.

Table 7 and Figure 4 represent the ROC analysis results for the higher G6PD. The AUC was statistically significant for NLR and SII. The AUC was 1.0 (95%CI: 1.0–1.0), with 100% sensitivity and 100% specificity and a cutoff value of lower than 0.3177 for NLR. Based on the data, 100% sensitivity and 98.6% specificity were established for NLR when it was equal to an integer (lower than 0.32). The AUCs were 0.893 (95%CI: 0.843–0.942) for SII, and sensitivity and specificity values were over 80%. 

## 4. Discussion

Prolonged jaundice is clinically detected jaundice that exceeds the physiologic jaundice period. Among the enzymopathies, G6PD and PK deficiencies are the most common defects in diverse ethnic groups [7,9]. Most of the prolonged jaundice in term newborns is caused by indirect hyperbilirubinemia, and a significant part of this is breast milk jaundice. Prolonged jaundice in newborns can sometimes be associated with underlying inflammation or infection [14,15]. The most important finding of this study was that G6PD had a perfect negative correlation with NLR, a strong negative correlation with SII, and a weak correlation with age and Hct. Furthermore, when adjusted for age and Hct, there was a weak negative correlation between G6PD and NLR and a moderate negative correlation between G6PD and SII. Neonates did not have obvious signs of hemolysis, such as anemia and reticulocytosis. A one-unit (about one standard deviation) increase in NLR reduces G6PD by 1.407. A 100-unit (almost one standard deviation) increase in SII reduces G6PD by 2.581. The explanatory power of the models is also very different. The model with NLR is 0.198; the model with SII is 0.346. Therefore, SII can be used as a better measure than NLR. The association between G6PD deficiency and markers of inflammation, such as NLR and SII, may suggest a potential link between inflammation and the pathophysiology of prolonged jaundice in term newborns with G6PD deficiency. 

Most newborn babies are clinically jaundiced. Jaundice is frequently mild, self-limiting, and generally accepted as physiologic. However, sometimes jaundice does not regress as expected, and prolonged jaundice may occur [16,17]. There are various factors in the etiology of prolonged jaundice and determination of the cause is important since the treatment, follow-up, and prognosis of each of them differ [18,19]. In the current study, all patients were breastfed, and 12 patients (7.2%) were also receiving formula. All the cases included in this study had prolonged jaundice due to breast milk. It has been reported that approximately one-third of breastfed babies are clinically jaundiced in the third week of life, and two-thirds of these babies have high indirect hyperbilirubinemia [20]. Although it was first suggested that pregnanediol, which is found in breast milk and is an inhibitor of hepatic glucuronyl transferase enzyme, may cause jaundice, it is currently accepted that it is not the primary factor but can contribute [20,21]. The mechanism that attracts the most attention is that an unidentified component of breast milk increases intestinal absorption of bilirubin. In addition, it has also been suggested that increased bilirubin absorption, rather than high bilirubin production, causes breast milk jaundice [20,22]. Although breast milk-induced jaundice may last up to the 16th week, it returns to normal in most babies during this period [20,23]. Although breast milk jaundice is a prolongation of normal physiologic jaundice, pathologic causes of jaundice should be ruled out if the bilirubin levels reach high values [20]. In our study, the diagnosis of breast milk jaundice was made after excluding other pathologic causes of jaundice. In previous studies, it was reported that serum bilirubin levels were 10–17 mg/dL in most breastfed infants [23,24], and the findings in our study are compatible with these studies. Therefore, other etiologies should be considered, especially in infants with elevated bilirubin levels above 17 mg/dL. In addition, hemograms, total, direct, and indirect bilirubin levels, and thyroid function tests should be evaluated to determine the etiology of bilirubin levels below 17 mg/dL. In breast milk jaundice, treatment is not started until bilirubin levels reach 20 mg/dL, and phototherapy may be started at bilirubin levels above 25 mg/dL [20]. Therefore, phototherapy was not initiated for the cases in our study. 

G6PD enzyme deficiency is the most common X-linked erythrocyte enzyme defect. In the context of prolonged jaundice, G6PD deficiency is relevant because it can predispose individuals to hemolysis (destruction of red blood cells), leading to an increase in bilirubin levels and the exacerbation of jaundice. The National Institute for Health and Clinical Excellence (NICE) recommends the analysis of direct bilirubin, urine culture, G6PD, CBC, and blood group in the evaluation of prolonged jaundice [16]. In the current study, it was determined that G6PD deficiency in infants led to the development of jaundice. There was no statistically significant difference in G6PD values according to gender, hospitalization status, formula use, ABO incompatibility, Rh incompatibility, and urine-reducing substance. These findings suggest that G6PD values were not significantly influenced by gender, hospitalization status, formula use, ABO incompatibility, Rh incompatibility, or the presence of specific urine-reducing substances in the study population. It is essential to consider these results in the context of the specific study population and the methodology used to ensure the reliability of the findings.

PK deficiency is an autosomal recessive enzyme deficiency that can be encountered in all populations and is less common than G6PD deficiency. In contrast to G6PD deficiency, reticulocytosis, jaundice, and anemia are present from the beginning. Jaundice can occur at levels high enough to require exchange transfusion. The enzyme deficiency is not only qualitative but is also due to a structural defect or lack of stabilization. PK deficiency should be considered in infants with negative Coombs tests and hemolytic anemia in case of prolonged jaundice without erythrocyte membrane defect [25,26,27,28]. In the current study, there was no statistically significant difference in PK values according to gender, hospitalization status, formula use, ABO incompatibility, Rh incompatibility, or urine-reducing substance. Overall, these findings suggest that PK values were not significantly influenced by these factors in the study population. There was no significant correlation between G6PD and PK activities. As one of the causes of prolonged jaundice, PK can result in indirect bilirubin due to increased reticulocyte elevation, which requires close follow-up. 

In recent years, inflammatory parameters, such as NLR, PLR, and SII, have been used as indicators in various diseases [29,30,31,32,33,34,35,36]. Karabulut et al. [30] suggested that NLR and mean platelet volume (MPV) may be used in addition to conventional parameters, such as CRP, in the diagnosis and subsequent management of early-onset neonatal sepsis. Kurt et al. [29] reported that the decrease in NLR and lymphocyte/monocyte ratio (LMR) after phototherapy could potentially be used in the evaluation of phototherapy’s effect on peripheral blood cells. Li et al. [31] demonstrated that there is a relationship between NLR and the presence of neonatal sepsis. Cakır et al. [32] suggested that the systemic inflammation response index (SIRI) may be a useful biomarker for predicting moderate to severe bronchopulmonary dysplasia (BPD) and a marker of clinical importance in the follow-up of infants with BPD. In the current study, although the CRP levels were within normal limits, G6PD had a strong negative correlation with NLR and SII. This implies that lower G6PD levels are associated with higher systemic inflammation, as reflected by an elevated SII. These correlations suggest that in individuals with jaundice, lower G6PD levels (indicating G6PD deficiency) are associated with higher levels of systemic inflammation, as measured by NLR and SII. This association may have clinical implications for managing jaundice, particularly in individuals with G6PD deficiency, as inflammation can exacerbate symptoms and complications associated with jaundice. Furthermore, when adjusted for age and Hct, there was a negative correlation among G6PD, NLR, and SII. Adjusting for age and hematocrit helps to control for potential confounding factors and allows for a more precise assessment of the relationship between G6PD levels and markers of systemic inflammation (NLR and SII) in the context of jaundice. These findings suggest that G6PD deficiency may contribute to increased systemic inflammation in individuals with jaundice, independent of age and Htc levels. According to ROC analysis, an NLR cutoff of 0.318 led to 100% sensitivity and 100% specificity, with an AUC of 1.0 (95%CI: 1.0–1.0). Based on the data, 100% sensitivity and 98.6% specificity were established for NLR when it was equal to an integer (lower than 0.32). The AUCs of 0.893 (95%CI: 0.843–0.942) for SII led to a sensitivity and specificity of 80%. However, it is essential to interpret these results cautiously and consider other potential confounders or factors that may influence the relationship between G6PD levels and markers of inflammation. Further research is needed to validate these findings and understand the underlying mechanisms linking G6PD deficiency and inflammation in individuals with jaundice. Although the findings in our study need to be supported by prospective randomized studies, we think that the use of NLR and SII as easily calculable methods in clinical practice may be useful in predicting prolonged jaundice. 

Prolonged jaundice is more common in male infants than female infants. In the current study, prolonged jaundice is more common in males than females. Siu et al. [37] reported that in total, 1164 infants (663 males, 501 females; male-to-female ratio = 1.3:1) with prolonged jaundice were referred to the Neonatal Jaundice Clinic (NNJC) during the 8-month study period. In total, 34 (5.13%) male infants and two (0.40%) female infants had G6PD deficiency. In a study conducted in Turkey, a total of 90 infants with prolonged jaundice were presented, including 50 male and 40 female neonates [38]. This is where our study showed similar results with males and females with G6PD deficiency [37,38,39]. The risk of developing significant prolonged jaundice is higher in male infants. This does not appear to be related to bilirubin production rates, which are similar to those in female infants. Male gender is one of the minor risk factors in prolonged jaundice.

G6PD is a regulatory enzyme of the pentose phosphate pathway that protects cells involved in NADPH production from oxidative stress. Therefore, cells are dependent on the G6PD enzyme for defense against oxidative damage. When the enzyme is deficient, oxidative stress and hemolysis of erythrocytes develop because glutathione cannot be reduced when cells are exposed to an oxidizing stress [40,41]. Consequently, during deficiency of this enzyme, there is increased oxidative stress, which results in increased inflammation in the body. Some studies have also reported that G6PD deficiency may be associated with a proinflammatory profile [42,43]. G6PD deficiency can be governed by many types of mutations, and the most common mutation in our study population would be G6PD Mediterranean [44,45]. This specific variant can cause severe G6PD deficiency, which in turn may cause a severe inflammatory response. In our study, there was a perfect negative correlation between NLR and G6PD (r: −1; *p* < 0.001; Figure 2). Although a mutation analysis of our cases was not performed, having the Mediterranean variant may more commonly explain this finding.

### Limitations of This Study

Selection bias is a potential limitation of our study. Formula-fed infants were not included in this study. Breast milk was not tested. This study is limited to infants diagnosed with hyperbilirubinemia in the neonatal unit of a private hospital in Istanbul. Since NLR and SII are inflammatory markers, although there is no evidence of this, an asymptomatic infection may have been present. Another possible confounding issue is nutrition. A mutation analysis of patients with G6PD enzyme deficiency was not studied. 

## 5. Conclusions

The mechanism of prolonged jaundice in otherwise healthy breastfed newborns is still unclear. Since prolonged jaundice may be the first sign of G6PD deficiency, the enzyme level should be checked in newborns diagnosed with this condition, and G6PD should be more strongly suspected over PK in prolonged neonatal jaundice due to breast milk jaundice. In communities in which G6PD deficiency is prevalent, hyperbilirubinemia can be detected early in cord blood, and neurotoxicity can be prevented. The explanatory power of the models is also very different. The model with NLR is 0.198; the model with SII is 0.346. Therefore, SII can be used as a better measure than NLR. The fact that these values are included in a routine test such as a CBC provides convenience in terms of use. Cases with elevated NLR and SII after birth should be followed up more closely, and it should be kept in mind that prolonged jaundice may develop.

## Figures and Tables

**Table 1:** Demographic and clinical characteristics of the patients.

	*n* Mean ± Std.	% Median (Q1–Q3)
Gender		
Male	106	63.5%
Female	61	36.5%
Age (days)	31 ± 6	30 (28–33)
Direct Cooms test		
Negative	167	100.0%
Urine Culture		
No reproduction	167	100.0%
Abdominal USG		
Normal	167	100.0%
Breast feeding		
Yes	167	100.0%
Formula		
No	155	92.8%
Yes	12	7.2%
ABO incompatibility		
No	157	94.0%
Yes	10	6.0%
Rh incompatibility		
No	152	91.0%
Yes	15	9.0%
Reducing substance (urine)		
No	140	83.8%
Yes	27	16.2%
Hospitalization		
No	160	95.8%
Yes	7	4.2%
Length of stay	2 ± 2	1 (1–1)
Day of onset of jaundice	2 ± 0	2 (1–2)
Time to fall below TB = 8 (days)	78±6	80 (80–80)
Gestational week	37±1	37 (36–38)

**Table 2:** Laboratory and clinical characteristics of patients.

	Mean ± Std.	Median (Q1–Q3)	Reference Values
WBC (10^3^ cells/µL)	9687 ± 2580	9110 (7910–11,100)	9.4–38
Hemoglobin (g/dL)	13.22 ± 2.27	13.1 (11.6–14.8)	13.4–19.8
Hct (%)	37.49 ± 6.7	37.1 (32.5–41.4)	41–65
Reticulocyte (%)	2.10 ± 0.80	1.93 (0.50–2.12)	0.5–2.5%
PLT (10^3^/µL)	344 ± 95	328 (277–400)	150–400
LYMPH (10^3^ cells/µL)	6.14 ± 1.57	5.98 (5.01–6.84)	2.8–9.1
NEU (10^3^ cells/µL)	2.04 ± 2.45	1.69 (1.2–2.22)	1.8–5.4
NLR	0.38 ± 0.91	0.3 (0.21–0.37)	
PLR	59.28 ± 22.28	55.38 (43.05–70.76)	
SII	114.88 ± 112.5	95.73 (64.61–125.96)	
MONO (10^3^ cells/µL)	1.31 ± 4.39	0.94 (0.7–1.27)	0–1.7
C–reactive protein (mg/L)	2.28 ± 1.42	2.3 (1.1–3.5)	>5
Sodium (mmol/L)	139.26 ± 2.99	139 (137–141)	136–145
Potassium (mmol/L)	4.53 ± 0.58	4.6 (4–5)	3.5–5.1
Urea (mg/dL)	8.3 ± 5.14	6.8 (4.6–12)	5–20
Creatinine (mg/dL)	0.45±0.34	0.42 (0.35–0.51)	Male: 0.74–1.35 Female: 0.59–1.04
Total biluribin (mg/dL)	12.54 ± 2.67	12.26 (10.86–14.21)	0.2–16.6
Direct biluribin (mg/dL)	0.60 ± 0.45	0.54 (0.48–0.62)	0.3–0.7
G6PD (U/g Hb)	21.95 ± 6.19	21.3 (17.11–25.63)	7.00–16.50
Pyruvate kinase (mU/10^9^ RBC/mL)	284.07 ± 91.56	278 (198–356)	111–406
fT4 (ng/dL)	1.13 ± 0.29	1.09 (1.01–1.19)	1.05–3.21
TSH (mU/L)	3.37 ± 1.88	3.14 (2.2–4.25)	0.73–4.77
AST (U/L)	39.11 ± 12.19	38 (31–47)	25–75
ALT (U/L)	23.80 ± 8.93	24 (17–36)	7–56
GGT (U/L)	110.50 ± 31.04	125 (88–136)	12–147
ALP (U/L)	250.67 ± 40.34	287 (248–315)	Male: 75–316 Female: 48–406
Protein (g/dL)	5.53 ± 0.59	5.6 (5.2–5.89)	6.0–8.3
Albumin (g/dL)	3.63 ± 0.56	3.6 (3.24–4.1)	1.90 to 4.90

WBC, white blood cell; Hb, hemoglobin; Hct, hematocrit; PLT, platelet; LYMPH, lymphocyte; NEU, neutrophil; NLR, neutrophil-to-lymphocyte ratio; PLR, platelet-to-lymphocyte ratio; SII, systemic immune inflammation index; MONO, monocyte; G6PD, glucose 6-phosphate dehydrogenase; fT4, free thyroxine; TSH, thyroid stimulating hormone; GGT, gamma-glutamyl transferase; ALP, alkaline phosphatase.

**Table 3:** Comparison of inflammatory markers (NLR, PLR, SII), G6PD activity, and PK activity according to some characteristics of patients.

	NLR	*p*	PLR	*p*	SII	*p*	G6PD	*p*	PK	*p*
Gender										
Male	0.3 (0.21–0.38)	0.286	56.61 (43.05–73.12)	0.854	97.7 (63.14–132.09)	0.379	21.22 (16.78–25)	0.288	265 (188–354)	0.071
Female	0.28 (0.18–0.35)		53.58 (44.15–70.24)		90.92 (65.69–114.08)		21.82 (18.23–27.56)		302 (231–358)	
Hospitalization										
No	0.3 (0.21–0.37)	0.701	56.61 (43.33–70.69)	0.539	95.81 (65.67–123.94)	0.917	21.27 (17.16–25.58)	0.701	279.5 (196.5–356)	0.358
Yes	0.21 (0.17–0.74)		50.11 (40.65–78.44)		76.17 (45.68–201.94)		25.45 (11.57–28.39)		245 (212–345)	
Formula										
No	0.3 (0.2–0.38)	0.862	55.35 (43.05–70.63)	0.406	94.69 (64.61–125.96)	0.488	21.23 (17.02–25.63)	0.865	280 (204–356)	0.095
Yes	0.26 (0.24–0.33)		61.7 (51.71–73.23)		108.31 (86.5–121.12)		22.68 (19.44–24.49)		243 (188–280.5)	
ABO incompatibility										
No	0.3 (0.21–0.37)	0.254	56.7 (43.81–70.76)	0.287	95.88 (67.35–125.96)	0.082	21.2 (17.11–25.55)	0.257	278 (195–356)	0.744
Yes	0.25 (0.15–0.32)		48.02 (40.03–62.84)		67.65 (47.27–111.85)		23.33 (20.44–29.72)		278 (235–354)	
Rh incompatibility										
No	0.3 (0.21–0.37)	0.991	55.37 (43.05–70.58)	0.455	95.34 (65.13–125.96)	0.878	21.25 (17.16–25.63)	0.989	274 (201–354)	0.437
Yes	0.28 (0.21–0.44)		58.74 (44.53–99.4)		99.17 (64.61–106.78)		21.82 (14.56–25.55)		312 (189–400)	
Urine reducing substance										
No	0.3 (0.21–0.36)	0.931	55.37 (42.88–70.69)	0.788	94.82 (63.87–124.67)	0.512	21.22 (17.24–25.63)	0.934	272 (189–355)	0.328
Yes	0.29 (0.23–0.39)		56.7 (44.56–75.42)		95.88 (71.54–131.06)		21.78 (16.74–24.65)		303 (228–356)	

**Table 4:** Correlation of G6PD activity and PK activity with NLR, PLR, SII, age, and HCT.

Variables	r-*p*	PK	NLR	PLR	SII	Age	Hct
**G6PD**	**r**	0.00	−1.000	−0.12	−0.837	0.296	−0.285
	***p***	0.960	**<0.001**	0.132	**<0.001**	**<0.001**	**<0.001**
**PK**	**r**		0.00	0.05	0.05	−0.04	−0.04
	***p***		0.968	0.501	0.510	0.617	0.613
**NLR**	**r**			0.12	0.837	−0.295	0.285
	***p***			0.132	**<0.001**	**<0.001**	**<0.001**
**PLR**	**r**				0.472	0.07	-0.290
	***p***				**<0.001**	0.340	**<0.001**
**SII**	**r**					−0.227	0.191
	***p***					**0.003**	**0.014**
**Age**	**r**						−0.286
	***p***						**<0.001**

**Table 5:** Results of partial correlation analysis of G6PD activity with NLR and SII adjusted for age and Hct.

		NLR	SII
**G6PD**	**Correlation coefficient (r)**	−0.212	−0.470
	***p* value**	0.006	<0.001

**Table 6:** Linear regression analysis results for G6PD activity.

Model	Unstandardized Coefficients (B)	95.0% Confidence Interval for B	*p* Value	R Square
**Model 1**				**0.198**
(Constant)	23.786	16.210/31.363	<0.001	
Age (days)	0.199	0.061/0.338	0.005	
HCT	−0.199	−0.340/−0.059	0.006	
NLR	−1.407	−2.411/−0.402	0.006	
**Model 2**				**0.346**
(Constant)	24.339	17.565/31.113	<0.001	
Age (days)	0.152	0.026/0.278	0.018	
HCT	−0.110	−0.238/0.018	0.091	
SII(100 unit)	−2.581	−3.331/−1.831	<0.001	

**Dependent variable:** G6PD; All models included age (days) and HCT. Model 1 includes the NLR, and Model 2 includes the SII.

**Table 7:** ROC analysis results for higher G6PD.

Variables	AUC	95%CI	*p* Value	Cutoff ^†^	Sensitivity	Specificity
NLR	1.0	1.0–1.0	<0.001	0.3177	100%	100%
NLR				0.32	100%	98.6%
SII	0.893	0.843–0.942	<0.001	100	83.2%	81.9%

^†^: values lower than the cutoff point.

## Data Availability

Participant-level data are available from the corresponding author.
